# Association of blood cadmium levels with epigenetic age acceleration in U.S. adults aged > 50 years

**DOI:** 10.3389/fpubh.2025.1504830

**Published:** 2025-04-15

**Authors:** Panpan Mi, Xu Cao, Haixia Feng, Huijie Wang

**Affiliations:** ^1^Department of Orthopedic, Hebei PetroChina Central Hospital, Langfang, China; ^2^Department of Endoscopy, Shijiazhuang Traditional Chinese Medicine Hospital, Shijiazhuang, China; ^3^Department of Tuberculosis, Shandong Public Health Clinical Center, Jinan, Shandong, China

**Keywords:** blood cadmium, epigenetic age, cross-sectional study, National Health and Nutrition Examination Survey, association

## Abstract

**Objectives:**

DNA methylation (DNAm) is a sensitive biomarker of aging-related processes, and novel epigenetic-based “clocks” can estimate accelerated biological aging. Cadmium (Cd) can alter cellular processes that promote aging and disrupt global methylation patterns. However, few studies have investigated the association between blood Cd and accelerated aging. We aimed to investigate the association between blood Cd and four DNAm-based epigenetic age accelerations in individuals over 50 in the United States, using data from the National Health and Nutrition Examination Survey (NHANES).

**Methods:**

DNAm-epigenetic biomarkers and blood Cd data from the NHANES database (1999–2002) were retrieved for this study. We evaluated four epigenetic ages: HorvathAge, HannumAge, PhenoAge, and GrimAge. Age acceleration was calculated by extracting the residuals from the regression of chronological age on each epigenetic age measure. We used weighted linear regression models and subgroup analyses to investigate the associations between blood Cd levels and these age accelerations, adjusting for potential confounding factors.

**Results:**

Higher blood Cd levels (≥0.5 μg/dl) were significantly associated with increased age acceleration for PhenoAge (β = 1.37, *P* = 0.017) and GrimAge (β = 1.31, *P* = 0.003) in adjusted models. A significant association was also observed for HannumAge (β = 0.94, *P* = 0.016), although this association was not significant for continuous Cd levels (*P* = 0.111). No significant associations were found for HorvathAge. Subgroup analyses indicated consistent associations across demographic and lifestyle subgroups, with no significant interactions.

**Conclusions:**

In this study, after adjusting for confounders, blood Cd levels were positively associated with PhenoAge acceleration and GrimAge acceleration in people over 50 in the United States. These results may be useful in proposing interventions in environmental exposures to slow the aging process and prevent age-related diseases.

## 1 Introduction

The aging population is a worldwide concern, leading to a heavy burden of chronic diseases and considerable social and economic expenses (1). However, chronological age is not a perfect indicator of biological aging (2). Individuals of the same chronological age can have significantly different risks for age-related diseases and mortality, which may reflect variations in their biological aging processes (3). DNA methylation (DNAm)-based epigenetic clocks have emerged as novel indicators of biological age and are correlated with mortality risk (4).

Increasing evidence suggests that several manifestations of aging are epigenetic (5–7). Epigenetic processes can result in lasting modifications to gene activity and phenotype, without changing the DNA sequence (8). DNAm, the most extensively studied epigenetic modification, typically involves the addition of a methyl group to cytosine-guanine dinucleotides (CpG sites). DNAm profiles reflect the influence of both external and internal factors (9). Oligonucleotide arrays covering extensive CpG sites, combined with mathematical algorithms, are used to assess DNAm age through epigenetic clocks (10, 11). Using DNAm age can uncover physiological variations among individuals who share the same chronological age. When DNAm age surpasses chronological age, it is referred to as epigenetic age acceleration (EAA) (12).

Furthermore, DNAm patterns are influenced by external stimuli (13). Cadmium (Cd) is a ubiquitous environmental pollutant known for its toxic effects on various biological systems (14–16). The main sources of Cd exposure include tobacco smoke, air pollution, occupational settings, and diet (leafy and root vegetables, grains, and organ meats) (17). Exposure to Cd has been identified as a major contributor to the development of various age-related diseases, such as osteoarthritis, cardiovascular diseases, and diabetes (18–21). Cd initiates age-related cellular processes, including gene-specific and global methylation disturbances (22). DNAm may be sensitive to Cd-related changes in oxidative stress (OS) (23, 24) and inflammatory environments (25). Studies have shown that Cd can competitively replace essential zinc ions in DNA methyltransferases (DNMTs), thereby inhibiting DNMT activity. This inhibition may lead to alterations in DNAm patterns, including global DNA hypomethylation and gene-specific hypermethylation at promoter regions (26). When specific CpG sites are affected, the DNAm age estimated from these sites could potentially change, which might indicate an impact of Cd exposure on biological aging.

Although numerous epigenetic clocks have been developed, HannumAge, HorvathAge, PhenoAge, and GrimAge stand out as widely recognized first- and second-generation epigenetic clocks (3, 10, 11, 27). HannumAge and HorvathAge were created by identifying sets of CpGs whose DNAm changed with chronological age (10, 11). PhenoAge is a distinct measure of biological age and is composed of age and nine clinical biomarkers (3). GrimAge is a composite marker that combines seven sets of CpGs to estimate the concentration of different plasma proteins (27). These clocks are distinguished by their robustness, extensive validation across diverse populations, and strong associations with various age-related diseases, highlighting their reliability in capturing biological aging processes. Due to the limited studies on the association between DNAm and Cd exposure in adults in the US, we conducted a cross-sectional study using the National Health and Nutrition Examination Survey (NHANES) database. This study aimed to assess the association between blood Cd levels and EAA using DNAm data.

## 2 Materials and methods

### 2.1 Study population

The NHANES is a nationwide survey that uses a complex multistage probability sampling design. Written informed consent was obtained from all participants, and the study protocol was approved by the National Center for Health Statistics Research Ethics Review Board. We selected the 1999–2000 and 2001–2002 cycles because DNAm-based epigenetic biomarkers for American adults were only available for these periods.

**Figure 1:**
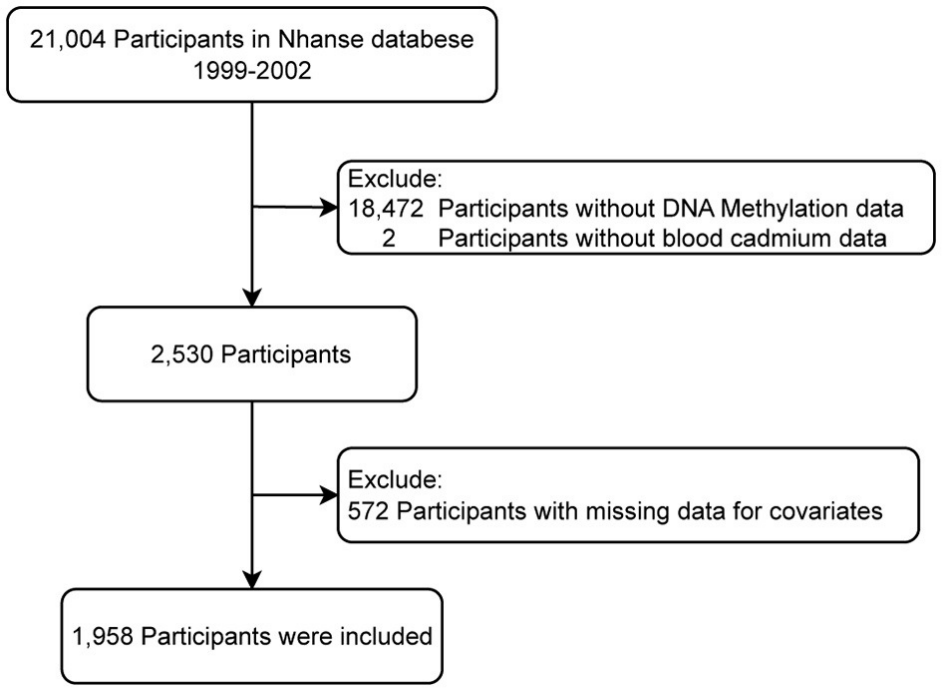
The study flowchart.

The study included 21,004 individuals from NHANES 1999–2002. Patients with missing data on epigenetic biomarkers (*n* = 18,472), blood Cd (*n* = 2), or covariates (*n* = 572) were excluded. Therefore, the final analysis included 1,958 eligible individuals (Figure 1).

### 2.2 Measurement of blood Cd levels

Blood Cd levels were measured using atomic absorption spectrometry with a PerkinElmer SIMAA 6,000 simultaneous multi-element atomic absorption spectrometer with Zeeman background correction.

### 2.3 DNAm—Epigenetic biomarkers

DNAm was measured from purified blood samples of these participants using the Illumina EPIC BeadChip. A methylation data matrix was generated, pre-processed, and normalized. DNAm-derived epigenetic biomarkers that predict chronological age, phenotypic age, telomere length, pace of aging, mortality, and mitotic cell turnover were identified. See the DNAm Arrays and Epigenetic Biomarkers data files for further details. Four biomarkers were selected: HorvathAge, HannumAge, PhenoAge, and GrimAge. Age acceleration was calculated by extracting residuals from the chronological age regression for each epigenetic age measure (28).

### 2.4 Covariates

The following covariates were included in the study. Chronological age (years) and body mass index (BMI, kg/m^2^) were treated as continuous variables and analyzed in their original scale without log transformation or normalization. Sex was classified as male or female; race was self-reported and classified into predefined categories (e.g., non-Hispanic White, non-Hispanic Black, Mexican American, and others). Smoking status was categorized into three groups: never, former, or current smoker. Alcohol consumption was dichotomized based on lifetime intake: fewer than 12 drinks or at least 12 drinks. Educational attainment was classified into three levels: <9 years, 9–12 years, and more than 12 years of education. Marital status was categorized as married or living with a partner, widowed/divorced/separated, or never married. Physical activity was assessed objectively and classified into four categories: mainly sitting, walking around, light load activities, or heavy load activities. Comorbidities were defined as self-reported physician-diagnosed diabetes, hypertension, stroke, or malignancy.

### 2.5 Statistical analysis

All models were analyzed in relation to accelerated epigenetic age, rather than chronological age, to better estimate the effects of blood Cd levels on biological aging. NHANES-recommended weights were used in this study. Continuous variables are described as mean (standard deviation) or median (interquartile range), whereas categorical variables are represented as proportions (%), as appropriate. Blood cadmium levels were analyzed both as continuous variables and as categorical variables (dichotomized at the median of 0.5 μg/l). Differences between groups were assessed using the χ^2^ test for categorical variables or by Student's *t*-test or Mann–Whitney *U*-test for continuous variables. The initial unadjusted model included blood Cd levels as the independent variable and the four age accelerations as dependent variables. The fully adjusted model included chronological age, sex, race, BMI, drinking and smoking status, highest degree obtained, marital status, and comorbidities as confounders. Additional subgroup analyses were performed to explore potential heterogeneity, and potential interactions were evaluated using likelihood ratio tests. All analyses were performed using R Statistical Software (version 4.2.2, http://www.R-project.org, The R Foundation) and Free Statistics analysis platform (version 2.0, Beijing, China) (29).

## 3 Results

This study included 1,958 participants, with a mean chronological age of 63.72 years. Participants were stratified into two groups based on blood Cd levels: < 0.5 μg/dl (*n* = 845) and ≥0.5 μg/dl (*n* = 1,113). The mean age of the low-Cd group was 62.62 years, whereas that of the high-Cd group was 64.62 years.

Table 1 summarizes the baseline characteristics of participants stratified by high and low blood Cd levels. Those with higher Cd levels were typically older (64.62 vs. 62.62 years), were more likely to be female (42.34% vs. 50.04% male), and had lower educational levels (28.89% with < 9 years vs. 20.48%). They also had lower poverty income ratios (median 2.67 vs. 3.77), lower BMI (27.97 vs. 29.37 kg/m^2^), and were more likely to be current smokers (26.30% vs. 2.26%). Additionally, higher Cd levels were associated with a higher prevalence of stroke (5.43% vs. 2.84%) and malignancy (20.36% vs. 12.90%).

**Table 1:** Participants characteristics by blood cadmium category, weighted^a^.

**Variables**	**Total *n* = 1,958**	**Blood cadmium**, μ**g/dl**		***P*-value**
		<**0.5** ***n*** = **845**	≥**0.5** ***n*** = **1,113**	
Age, year	63.72 (10.17)	62.62 (9.68)	64.62 (10.47)	0.001
Sex, male	1,005 (45.80)	456 (50.04)	549 (42.34)	0.003
**Race**				
Mexican American	546 (3.38)	223 (3.27)	323(3.48)	0.410
Other Hispanic	118 (5.42)	53 (5.85)	65 (5.07)	
Non-Hispanic white	834 (80.23)	364 (80.26)	470 (80.21)	
Non-Hispanic black	395 (7.77)	182 (8.12)	213 (7.48)	
Others	65 (3.20)	23 (2.50)	42 (3.77)	
**Education level, year**				
< 9	848 (25.12)	331 (20.48)	517 (28.89)	0.004
9–12	416 (27.34)	176 (25.88)	240 (28.52)	
>12	694 (47.55)	338 (53.64)	356 (42.59)	
**Marital status**				
Married or living with a partner	1,275 (68.95)	598 (75.69)	677 (63.46)	< 0.001
Widowed/divorced/separated	610 (27.88)	213 (21.54)	397 (33.05)	
Never married	73 (3.17)	34 (2.77)	39 (3.49)	
Poverty income ratio	3.18 (1.63, 5.00)	3.77 (1.89, 5.00)	2.67 (1.38, 5.00)	< 0.001
BMI, kg/m^2^	28.60 (6.04)	29.37 (6.32)	27.97 (5.73)	0.007
**Physical activities**				
Mainly sit	531 (27.18)	193 (22.42)	338 (31.05)	0.026
Walk around	1,128 (55.06)	522 (59.68)	606 (51.30)	
Light load	233 (14.60)	101 (15.08)	132 (14.22)	
Heavy load	66 (3.16)	29 (2.83)	37 (3.43)	
**Smoking status**				
Never	903 (44.02)	521 (58.83)	382 (31.95)	< 0.001
Former	776 (40.47)	311 (38.90)	465 (41.75)	
Current	279 (15.51)	13 (2.26)	266 (26.30)	
**Drinking status**				
No	336 (15.28)	164 (16.55)	172 (14.24)	0.450
Yes	1,622 (84.72)	681 (83.45)	941 (85.76)	
Hypertension	1,206 (56.98)	520 (57.59)	686 (56.48)	0.692
Diabetes	457 (16.47)	221 (17.67)	236 (15.50)	0.362
Stroke	98 (4.26)	40 (2.84)	58 (5.43)	0.008
Malignancy	274 (17.01)	96 (12.90)	178 (20.36)	0.001

BMI, body mass index.

^a^Data are presented as unweighted number (weight percentage) for categorical variables, weighted mean (standard deviation) or weighted median (Q1, Q3) for continuous variables.

Table 2 presents the epigenetic age characteristics by blood Cd levels in a weighted sample of 1,958 participants. The table demonstrates that higher blood Cd levels (≥0.5 μg/dl) are associated with significantly higher epigenetic ages across all measures (HorvathAge, HannumAge, PhenoAge, and GrimAge) compared to lower Cd levels (< 0.5 μg/dl), with *P*-values < 0.001. For age acceleration metrics, significant differences were observed in PhenoAge (*P* < 0.001), GrimAge (*P* < 0.001), and HannumAge (*P* < 0.001), while HorvathAge showed a marginally significant difference (*P* = 0.035). See Supplementary Table 1 for unweighted analyses and Supplementary Figure 1 for the distributions of EAA metrics.

**Table 2:** Epigenetic age characteristics by blood cadmium category, weighted^a^.

**Variables**	**Total *n* = 1,958**	**Blood cadmium**, μ**g/dl**		***P*-value**
		<**0.5** ***n*** = **845**	≥**0.5** ***n*** = **1,113**	
**Epigenetic age**				
HorvathAge	65.26 (9.20)	64.20 (8.76)	66.12 (9.46)	< 0.001
HannumAge	64.85 (9.88)	63.34 (9.41)	66.09 (10.08)	< 0.001
PhenoAge	53.38 (10.93)	51.32 (10.56)	55.06 (10.95)	< 0.001
GrimAge	64.18 (8.93)	61.51 (8.30)	66.35 (8.83)	< 0.001
**Age acceleration**				
HorvathAge	0.12 (5.05)	−0.10 (4.93)	0.31 (5.15)	0.035
HannumAge	−0.28 (5.08)	−0.90 (4.93)	0.23 (5.15)	< 0.001
PhenoAge	−0.30 (6.49)	−1.43 (6.21)	0.63 (6.56)	< 0.001
GrimAge	−0.26 (4.86)	−2.10 (3.51)	1.25 (5.28)	< 0.001

^a^Data are presented as weighted mean (standard deviation).

We conducted further linear regression analyses to explore the relationship between blood Cd levels and EAAs (Table 3). Higher blood Cd levels (≥0.5 μg/dl) were significantly associated with increased age acceleration in PhenoAge and GrimAge, both in non-adjusted and adjusted models. Specifically, in the adjusted models, participants with blood Cd levels ≥0.5 μg/dl exhibited greater PhenoAge acceleration (β = 1.37, 95% CI: 0.34–2.40, *P* = 0.017) and GrimAge acceleration (β = 1.31, 95% CI: 0.65–1.96, *P* = 0.003) compared to those with lower Cd levels (< 0.5 μg/dl). Similarly, continuous blood Cd levels were positively associated with PhenoAge acceleration (β = 1.36, 95% CI: 0.16–2.56, *P* = 0.032) and GrimAge acceleration (β = 2.14, 95% CI: 1.19–3.09, *P* = 0.002) after adjusting for covariates. For HannumAge, higher blood Cd levels (≥0.5 μg/dl) were significantly associated with increased age acceleration in the adjusted model (β = 0.94, 95% CI: 0.25–1.64, *P* = 0.016), although the association with continuous Cd levels was not statistically significant (β = 0.78, 95% CI: −0.24–1.80, *P* = 0.111). In contrast, no significant associations were observed between blood Cd levels and HorvathAge acceleration in either continuous or categorical analyses (all *P* > 0.05).

**Table 3:** Linear regression models between blood cadmium with epigenetic age acceleration, weighted.

**Variable**	**Non-adjusted Model** ^**a**^		**Adjusted Model** ^**b**^	
	β **(95% CI)**	***P*** **-value**	β **(95% CI)**	***P*** **-value**
**HorvathAge**				
Blood Cd (continuous)	0.25 (−0.27, 0.77)	0.336	0.14 (−0.67, 0.94)	0.694
Blood Cd ≥0.5 μg/dl	0.41 (0.03, 0.79)	0.035	0.34 (−0.23, 0.92)	0.192
**HannumAge**				
Blood Cd (continuous)	0.90 (0.08, 1.72)	0.032	0.78 (−0.24, 1.80)	0.111
Blood Cd ≥0.5 μg/dl	1.13 (0.56, 1.69)	< 0.001	0.94 (0.25, 1.64)	0.016
**PhenoAge**				
Blood Cd (continuous)	2.02 (0.79, 3.24)	0.002	1.36 (0.16, 2.56)	0.032
Blood Cd ≥0.5 μg/dl	2.06 (1.16, 2.96)	< 0.001	1.37 (0.34, 2.40)	0.017
**GrimAge**				
Blood Cd (continuous)	4.68 (3.28, 6.08)	< 0.001	2.14 (1.19, 3.09)	0.002
Blood Cd ≥0.5 μg/dl	3.35 (2.56, 4.14)	< 0.001	1.31 (0.65, 1.96)	0.003

Cd, cadmium; BMI, body mass index.

^a^No covariates adjusted.

^b^Adjusted for sex, age, BMI, race, smoking status, drinking status, marital status, education level, physical activity, poverty income ratio, diabetes, stroke, malignancy, and hypertension.

**Figure 2:**
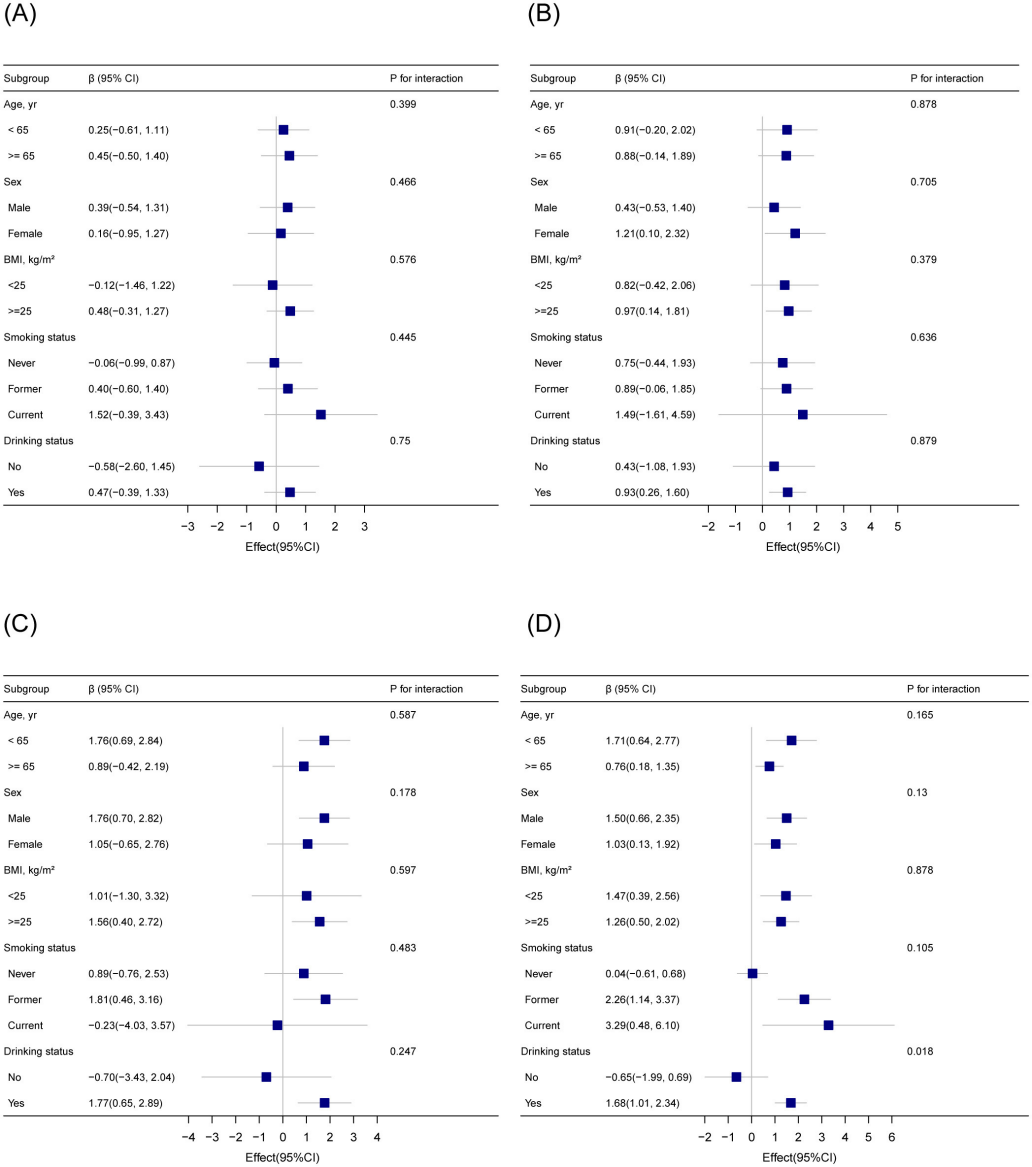
Subgroup analysis of the association between blood cadmium levels (binary) and epigenetic age acceleration based on DNA methylation **(A)** HorvathAge; **(B)** HannumAge; **(C)** PhenoAge; **(D)** GrimAge. Adjusted for sex, age, body mass index, race, smoking status, drinking status, marital status, education level, physical activity, poverty income ratio, diabetes, stroke, malignancy, and hypertension.

Subgroup analyses were conducted to examine whether the association between blood cadmium levels (binary: < 0.5 μg/dl vs. ≥0.5 μg/dl) and EAAs (Figure 2). The results indicated that the association was consistent across subgroups defined by age, sex, BMI, and smoking status, with no significant interactions observed (*P* for interaction >0.05 for all subgroups). For drinking status, the association between blood cadmium levels and GrimAge acceleration showed a nominal *P*-value of 0.018. However, after applying multiple test corrections, this interaction was no longer statistically significant. These findings suggest that the relationship between blood Cd levels and EAAs is generally stable across various demographic and lifestyle subgroups, with no robust evidence of effect modification by the factors examined.

## 4 Discussion

This cross-sectional study examined the association between blood Cd levels and four DNAm-based EAAs in adults aged >50 years in the United States. Among the 1,958 participants, higher blood Cd levels were associated with PhenoAge and GrimAge acceleration, after adjusting for demographic factors and comorbidities. A subgroup analysis confirmed the stability of these associations.

Aging is associated with a global decrease in DNAm and a localized increase in methylation at CpG islands and specific promoters (30–32). Increasing evidence suggests numerous links between age and DNAm, leading to the development of molecular epigenetic clocks that represent the biological age (33, 34). These clocks have been developed using different methodologies and have varying levels of evidence supporting their validity as biomarkers of age-related health decline (28). PhenoAge (3) and GrimAge (27), which are second-generation clocks, outperformed HorvathAge and HannumAge in predicting health decline and mortality (12, 27, 35, 36). GrimAge stands out as the most powerful epigenetic indicator of mortality because it is uniquely crafted to forecast adult mortality (27). The inconsistent associations between blood Cd levels and different epigenetic clocks may be attributed to the differences in the design and sensitivity of these clocks. First-generation clocks may be less effective in detecting age-related decline because they do not incorporate clinical biomarkers in their derivation. As a result, they might be less sensitive to capturing EAA caused by external exposures, particularly HorvathAge, which is most closely related to chronological age. This could explain the lack of significant associations with HorvathAge in our study. Additionally, DNAmHannum is based on 71 CpG sites, while DNAmHorvath includes 353 CpGs, DNAmPhenoAge includes 513 CpGs, and DNAmGrimAge includes 1,030 CpGs. The number of CpG sites included in these clocks may influence their sensitivity to reflect underlying aging processes and biological responses to Cd exposure. These factors may explain why blood Cd levels were not associated with HorvathAge acceleration but were positively associated with HannumAge, PhenoAge, and GrimAge acceleration.

Few studies have examined the association between epigenetic acceleration and Cd exposure. Before the release of the NHANES DNAm data, Zhang et al. (37) calculated PhenoAge using the NHANES database and found a positive association between urinary Cd levels and PhenoAge in fully adjusted models [2.13 years per 1 ng/g urinary Cd, (1.67, 2.58)]. However, the relationship between Cd exposure and PhenoAge acceleration has not been investigated. Notably, a study investigating the relationship between Cd levels and HorvathAge and HannumAge in non-smoking women in northern Thailand found that the high urinary Cd group had lower HorvathAge and HannumAge accelerations than the low urinary Cd group, contrary to the expected age acceleration effect (22). In our results, blood Cd levels were not significantly associated with HorvathAge acceleration or HannumAge acceleration, and smoking status did not affect the association between blood Cd levels and PhenoAge or GrimAge acceleration.

However, mechanisms underlying the association between Cd exposure and EAA remain unclear. Cd toxicity is believed to occur through mechanisms such as OS, DNA damage, and cell death (38), which are associated with telomere shortening, a key mechanism of cellular and organismal aging (39–42). Experimental studies have shown that Cd promotes OS by catalyzing the production of reactive oxygen species and interfering with antioxidant responses (43, 44). Additionally, Cd can trigger the release of inflammatory cytokines (45). Inflammation, in turn, may hasten the shortening of leukocyte telomeres by enhancing cell turnover, promoting replicative senescence, and causing OS (46). When telomeres reach a critically short length, cellular senescence is initiated, leading to a loss of the cell's ability to divide (47).

The strengths of this study include the use of four epigenetic age measures, which provide a comprehensive assessment of EAA. This approach enhanced the robustness and credibility of our results when analyzing the association between blood Cd levels and EAA. Additionally, we used the NHANES database, which employs a sophisticated multistage probability sampling design, to ensure the selection of a representative sample from a non-institutionalized civilian population. Consequently, extrapolating our weighted results to the entire non-institutionalized civilian population of the United States is considered highly reliable.

This study has several limitations. First, the cross-sectional nature restricted our ability to establish a causal relationship between blood Cd levels and the acceleration of epigenetic age. Longitudinal studies are required to establish the temporal relationships and causality. Second, the study population was limited to a specific demographic group, which may have affected the generalizability. Third, although we adjusted for several potential confounders, residual confounding factors could not be entirely ruled out. Finally, the measurement of blood Cd levels at a single time point may not accurately reflect the long-term exposure. Repeated measurements over an extended period would provide a more comprehensive assessment of Cd exposure.

## 5 Conclusion

This study revealed that higher blood Cd levels were positively associated with HannumAge, PhenoAge, and GrimAge acceleration in U.S. adults aged > 50 in the United States, even after adjusting for confounders. These findings indicate that Cd exposure could potentially impact the biological aging process in a measurable way. Further investigation is warranted in the future to examine longitudinal Cd exposure and biological aging outcomes.

## Data Availability

The raw data supporting the conclusions of this article will be made available by the authors, without undue reservation.

## Supplementary material

Supplementary Figure 1 Distribution of epigenetic age acceleration metrics.

Supplementary Table 1 Unweighted analysis of epigenetic age acceleration by blood cadmium levels.
