# Supplementary figures and images for: Association of blood cadmium levels with epigenetic age acceleration in U.S. adults aged > 50 years
Source: Front Public Health. 2025 Apr 15;13:1504830. doi: 10.3389/fpubh.2025.1504830 (PMC12037496; doi:10.3389/fpubh.2025.1504830)

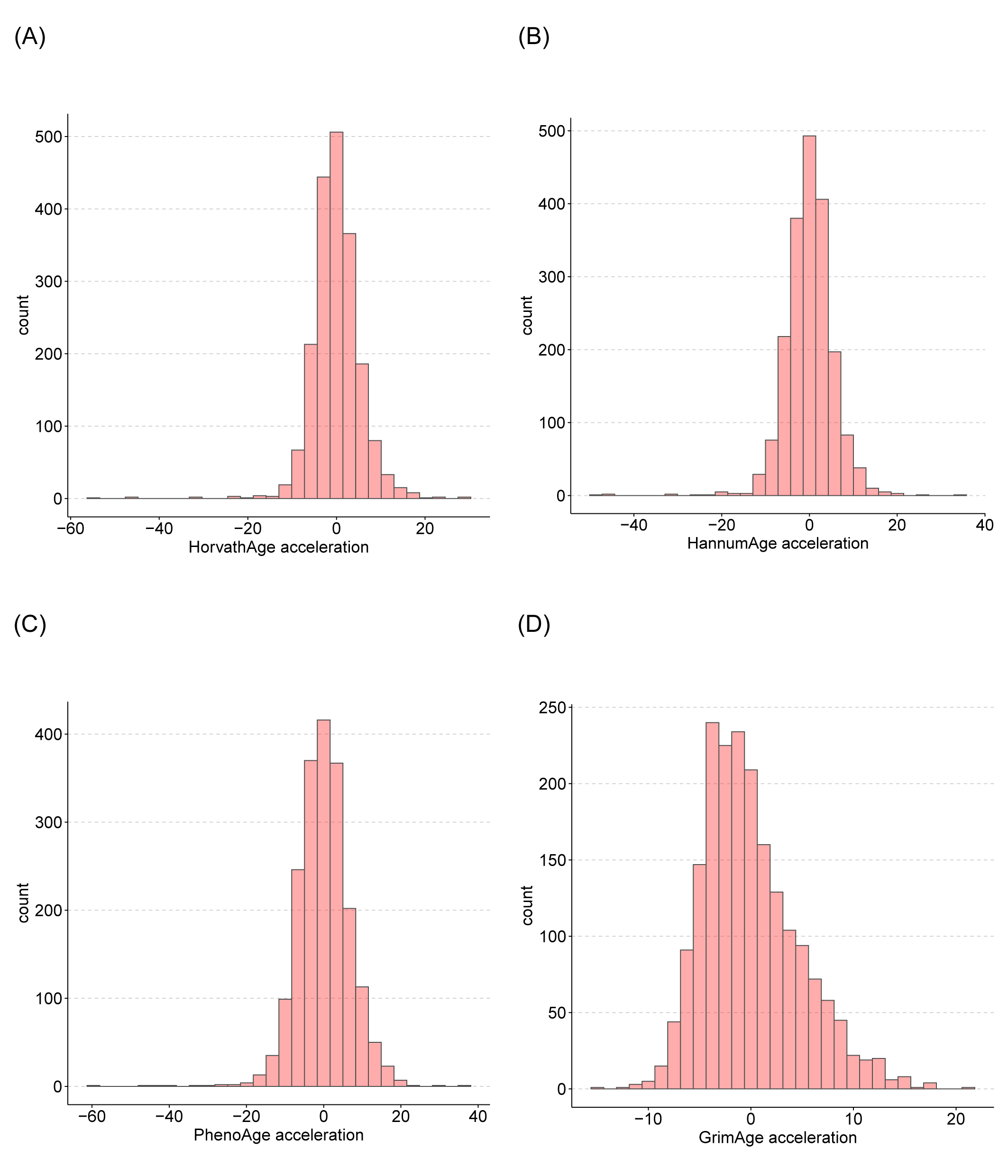

Supplement: Supplementary Figure 1 — Distribution of epigenetic age acceleration metrics. [file Image_1.TIF]
