# Supplementary material for: Association of blood cadmium levels with epigenetic age acceleration in U.S. adults aged > 50 years
Source: Front Public Health. 2025 Apr 15;13:1504830. doi: 10.3389/fpubh.2025.1504830 (PMC12037496; doi:10.3389/fpubh.2025.1504830)
Supplement: Supplementary Table 1 — Unweighted analysis of epigenetic age acceleration by blood cadmium levels. [file Table_1.DOCX]

**Table S1.** Unweighted analysis of epigenetic age acceleration by blood cadmium levels.

| Variable | Total (n = 1958) | Blood cadmium, ug/dL | | *P*-value |
| --- | --- | --- | --- | --- |
|  |  | <0.5  n = 845 | ≥0.5  n = 1,113 |  |
| HorvathAge | 0.0 ± 5.5 | -0.2 ± 5.4 | 0.2 ± 5.6 | 0.092 |
| HannumAge | 0.0 ± 5.5 | -0.5 ± 5.4 | 0.4 ± 5.6 | < 0.001 |
| PhenoAge | 0.0 ± 6.9 | -1.0 ± 6.6 | 0.8 ± 7.0 | < 0.001 |
| GrimAge | 0.0 ± 4.7 | -1.6 ± 3.5 | 1.2 ± 5.2 | < 0.001 |

Data are presented as mean ± standard deviation.
